# Prognostic factors of a favorable outcome following a supervised exercise program for soldiers with sub-acute and chronic low back pain

**DOI:** 10.1186/s12891-018-2022-x

**Published:** 2018-04-02

**Authors:** Marc Perron, Chantal Gendron, Pierre Langevin, Jean Leblond, Marianne Roos, Jean-Sébastien Roy

**Affiliations:** 1 Department of Rehabilitation, Faculty of Medicine, Université Laval, Pavillon Ferdinand-Vandry, Local 4445, 1050, avenue de la Médecine, Québec, QC G1V 0A6 Canada; 2 Canadian Forces Health Services Group, Valcartier Garison, Quebec City, Canada; 3 Physio Interactive, Quebec City, Canada; 4 Centre for Interdisciplinary Research in Rehabilitation and Social Integration (CIRRIS), Quebec City, Canada

**Keywords:** Low back pain, Clinical prediction rule, Exercises

## Abstract

**Background:**

Low back pain (LBP) encompasses heterogeneous patients unlikely to respond to a unique treatment. Identifying sub-groups of LBP may help to improve treatment outcomes. This is a hypothesis-setting study designed to create a clinical prediction rule (CPR) that will predict favorable outcomes in soldiers with sub-acute and chronic LBP participating in a multi-station exercise program.

**Methods:**

Military members with LBP participated in a supervised program comprising 7 stations each consisting of exercises of increasing difficulty. Demographic, impairment and disability data were collected at baseline. The modified Oswestry Disability Index (ODI) was administered at baseline and following the 6-week program. An improvement of 50% in the initial ODI score was considered the reference standard to determine a favorable outcome. Univariate associations with favorable outcome were tested using chi-square or paired t-tests. Variables that showed between-group (favorable/unfavorable) differences were entered into a logistic regression after determining the sampling adequacy. Finally, continuous variables were dichotomized and the sensitivity, specificity and positive and negative likelihood ratios were determined for the model and for each variable.

**Results:**

A sample of 85 participants was included in analyses. Five variables contributed to prediction of a favorable outcome: no pain in lying down (*p* = 0.017), no use of antidepressants (*p* = 0.061), FABQ work score < 22.5 (p = 0.061), fewer than 5 physiotherapy sessions before entering the program (*p* = 0.144) and less than 6 months’ work restriction (*p* = 0.161). This model yielded a sensitivity of 0.78, specificity of 0.80, LR+ of 3.88, and LR- of 0.28. A 77.5% probability of favorable outcome can be predicted by the presence of more than three of the five variables, while an 80% probability of unfavorable outcome can be expected if only three or fewer variables are present.

**Conclusion:**

The use of prognostic factors may guide clinicians in identifying soldiers with LBP most likely to have a favorable outcome. Further validation studies are needed to determine if the variables identified in our study are treatment effect modifiers that can predict success following participation in the multi-station exercise program.

**Trial registration:**

ClinicalTrials.gov Identifier: NCT03464877 registered retrospectively on 14 March 2018.

**Electronic supplementary material:**

The online version of this article (10.1186/s12891-018-2022-x) contains supplementary material, which is available to authorized users.

## Background

Attempts to treat low back pain (LBP) have typically had limited success [1–4]. These difficulties are believed to be due, in part, to the fact that the cause of the pain is rarely known, leaving approximately 85% of individuals with LBP to be included in a “non-specific LBP” group [4, 5]. However, LBP is a complex condition influenced by a range of psychosocial, physical and activity-related factors that differ from person to person [6–9], rendering the non-specific LBP group heterogeneous, and unlikely to respond to the same treatment approach. It has thus been suggested that classification of LBP subjects into subgroups, followed by specific treatment of each subgroup according to their needs, is necessary in order to efficiently treat this condition [9]. Different classification systems, in which LBP subjects are assigned according to personal and clinical characteristics, have therefore been created and modified over the past 20 years [10, 11]. However, more conclusions on how to classify LBP sufferers and how to treat each subgroup are needed [12, 13]. Recent literature reviews still examine studies implementing interventions in non-specific, unclassified LBP populations. The most recent reviews evaluating the efficiency of exercise programs in the treatment of chronic LBP, for example, did not discuss LBP subgroups but treated the subjects as a homogenous population [4, 14]. Searle et al.’s review [4] confirmed the previously-held belief that stabilization and strengthening exercise programs decrease chronic LBP, but with only small effects. It remains to be examined whether division of the LBP population into subgroups will allow researchers and clinicians to obtain larger treatment effects when using exercise program interventions [2, 4, 15].

In order to accurately classify LBP patients into subgroups to which a specific intervention is assigned, valid clinical prediction rules (CPR) need to be developed [16, 17]. These rules are sets of data obtained from the history and physical evaluation of each patient, as well as from auto-administered questionnaires, that indicate which subgroup of patients will likely respond to a specific intervention. In 2011, a multi-station full-body supervised exercise program was created for soldiers with LBP and implemented at a military base of the Canadian Armed Forces. Although the program has enjoyed some clinical success, as subjectively reported by a number of soldiers, it is of great importance to maximize the efficacy of the program by determining the LBP subgroup most susceptible to benefit from it.

The development of CPR typically follows a three-step process: derivation, validation and impact analysis [16, 18, 19]. Derivation is the early hypothesis generation step in which prognostic factors are identified from a set of clinical variables that are believed to have predictive value, based on clinical experience or previous research. Kent et al. [20] recommended subdividing the derivation step into two sub-steps: hypothesis-setting, which represents the exploratory phase in which data from cohort studies may generate hypotheses about potential treatment effect modifiers, and hypothesis-testing, which is the confirmation sub-step in which pre-specified (a priori) hypotheses are tested more rigorously on subgrouping effects in samples of people similar to those who participated in the hypothesis-setting study. Subsequently, a validation step is required to identify treatment effect modifiers by testing subgroups/treatment interaction effects in randomized controlled trials involving samples of people with characteristics that are similar to (narrow validation) or different from (broad validation) those in the hypothesis-testing study. Lastly, the impact analysis aims to verify the usefulness of the CPR in improving outcomes and patient satisfaction and in decreasing costs once it is implemented in clinical practice.

According to this development framework, the present study is at the early hypothesis-setting step. Our objective was therefore to identify variables associated with a favorable outcome in soldiers with sub-acute and chronic LBP participating in a multi-station full-body supervised exercise program. The results obtained may permit generation of potential treatment effect modifiers that will eventually have to be validated before being recommended for clinical practice.

## Methods

### Participants

Military members consulting at Valcartier Health Centre for LBP were consecutively recruited. To be included, potential participants had to be aged 18 and older and present with an episode of subacute or chronic LBP with or without radiation to the lower limbs. Potential participants were also required to have a minimal score of 17% on the Modified Oswestry Disability Index (ODI) at the initial evaluation (based on the clinically important difference of this test [21]). Patients were excluded in the following circumstances: previous surgery to the spinal column, lumber spine injection in the past two weeks, signs of upper motor neuron lesions (bilateral paresthesia, hyperreflexia or spasticity), serious medical conditions (e.g. tumor, fracture, rheumatoid arthritis, osteoporosis) and unavailability to participate in the 6-week exercise program. Patients admitted to the clinic with acute LBP (e.g. onset of constant and intense pain [> 5/10] < 7 days, severely limited lumbar range of motion [more than 50% in at least 2 directions], obvious lateral shift) were first treated by their physiotherapist and then referred to the project coordinator, once the risk of harm associated with participation in the program was deemed low (e.g. when the indicators of acute LBP were no longer present). Participation in the study was voluntary, and informed consent forms were signed by all subjects. This study was approved by the ethics committee of the CIUSSS de la Capitale-Nationale (Quebec Rehabilitation Institute).

Previous CPR developed for rehabilitation programs in LBP populations [15, 22, 23] included 5 predictors or fewer in their final model. According to the formula suggested by Green [24] for regression analysis (50 + [8 x number of prognostic factors]), and considering the expected 5 variables in the final model, as well as an estimated 20% dropout rate, the ideal sample size was 108.

### Study design

All participants took part in the 6-week exercise program, as well as in the two evaluation sessions (pre- and post- exercise program). At the initial evaluation, subjects completed forms and questionnaires on sociodemographics, symptomatology, comorbidities, work restrictions, pain and functional limitations and fear-avoidance beliefs. A physiotherapist measured their lumbar and hip mobility, conducted diagnostic and pain provocation tests and assessed endurance of the trunk muscles. Following the initial evaluation, subjects took part in the 6-week multi-station full-body supervised exercise program (2 to 3 sessions per week). The ODI was completed at the initial and at the final evaluations. The change in ODI score following the program was considered the principal measure reflecting favorable or unfavorable outcome.

### Evaluations and variables

The initial evaluation was carried out by four experienced physiotherapists who completed a three-hour training session in order to standardize the evaluation protocol. Selection of the variables included in the clinical examination was based on the results of previous studies that aimed to develop preliminary CPR in patients with LBP [15, 22, 23]. The following clinical variables were collected.

#### Standardized subjective clinical questionnaire

This questionnaire documented participants’ personal and occupational characteristics, personal medical history, current and past episodes of LBP, a detailed description of their symptoms (low back and lower limb pain mapping), pain behaviour (aggravating or relieving factors such as sitting, bending, standing, supine, walking, lifting) [25] and other characteristics such as work restrictions and number of treatments received before the initial evaluation.

#### Modified Oswestry disability index

The ODI is a self-administered questionnaire whose purpose is to evaluate the severity of the limitations and restrictions suffered by patients with LBP. It consists of 10 items that assess the interference of LBP with activities of daily living. Each item is scored on a categorical scale from 0 to 5, with higher values representing greater disability. The score out of 50 is multiplied by two and expressed as a percentage. The reliability (ICC = 0.86), construct validity and sensitivity to change of this questionnaire have previously been demonstrated [26]. The minimal detectable change in persons with non-specific LBP is 10 points [21, 27, 28], while the minimal clinically important difference ranges from 10 to 19 [21, 26, 29]. Patients who experienced an improvement of at least 50% on the ODI were categorized as having a favorable outcome [15, 22, 23].

#### Fear-avoidance beliefs questionnaire (FABQ)

The FABQ is a self-administered questionnaire that consists of 16 questions pertaining to patients’ beliefs regarding the effect of their physical activities and work on their LBP [30]. It comprises a physical activity subscale (5 questions) and a work subscale (11 questions). Each item is scored from 0 to 6, with higher scores indicating higher levels of fear-avoidance behavior. Moderate to high test-retest reliability was found respectively for the Physical Activity subscale (ICC = 0.72 to 0.90) and the Work subscale (ICC = 0.8 to 0.91) [31].

#### Numeric pain rating scale (NPRS)

This tool was used to document the worst and average LBP experienced by participants in the 48 h preceding the initial evaluation. Pain intensity was rated on a scale from 0 to 10, where 10 is the worst pain imaginable. Its reliability is moderate (ICC = 0.76) [32].

#### Lumbar and hip mobility

Lumbar range of motion (ROM) was evaluated by measuring the distance between the third fingertip and the floor in trunk forward and lateral flexions (ICC = 0.91–0.99) [33]. The single inclinometer method was used to measure trunk extension (ICC = 0.61–0.95) [34]. Passive hip ROM was measured bilaterally using a goniometer or an inclinometer (ICC = 0.56–0.99) [35] and included internal rotation, external rotation, flexion and extension.

#### Signs of instability

Aberrant lumbar movement patterns during forward flexion that are believed to be associated with instability were documented. These patterns include a painful arc, Gower’s sign (thigh climbing), brisk movement (instability catch) or an inverted lumbar pelvic rhythm [36].

The response to the following clinical screening, diagnostic or performance tests was also documented: 1) The Straight Leg Raise, used to screen for herniated discs and to verify neural sensitivity (ICC = 0.93–0.97) [37]. The amplitude of the leg raise was measured with an inclinometer placed on the subject’s tibia. 2) The Biering-Sorensen, McGill and lateral plank tests, designed to measure muscle endurance in trunk extension, flexion and lateral flexion (ICC = 0,89–0.98) [33], respectively. 3) The Prone Instability Test, used to identify patients with lumbar instability, and which has been shown to have predictive value for the stabilization group of the Treatment-Based Classification (TBC) [11] (ICC = 0.74–1.00) [36]. 4) Central posterior to anterior pressure techniques were executed to evaluate pain provocation and segmental mobility of the lumbar spine in prone position. Segment mobility was classified as hypomobile, normal or hypermobile by applying a posterior to anterior force on the spinous process of each lumbar vertebra with the hypothenar eminence. Pain was rated as present or absent. The interrater reliability of this technique is good for the identification of the least mobile segment (agreement = 82.8%, kappa = 0.71, 95% CI = 0.48 to 0.94), but poor for determining the most mobile segment (kappa = 0.29, 95% CI = − 0.13 to 0.71) [33]. The reliability of this procedure seems higher to assess pain provocation than segmental mobility [38].

### Intervention

Each participant took part in the 6-week multi-station full-body supervised exercise program, which consisted of two to three sessions per week (45 to 60 min each) and was supervised by a physiotherapist. The exercise program is composed of 7 stations, each consisting of numerous exercises of increasing difficulty. The exercises were grouped together as follows: Hip strengthening and control (Station 1); The squat and its variants (Station 2); Elastic bands and the Bodyblade (Station 3); Abdominal planks and their variants (Station 4); Abdominal strengthening (Station 5); Back extensor strengthening (Station 6); and Lifting techniques (Station 7). Each of the exercises in the program is shown in an Additional file 1. The basic principal applied for all exercises was the maintenance of natural lordosis, regardless of the weight or external forces imposed on the body. Exercise parameters were chosen to increase strength, endurance and neuromuscular control. In accordance with recognized motor learning principles [39], participants were encouraged to complete a large variety of exercises and to focus on the quality, rather than the quantity, of their movements. Progression in the program led to the execution of exercises that simulated functional and occupational tasks (task-oriented approach). The initial difficulty level and choice of exercises were determined by the supervising physiotherapist according to 3 principal criteria: severity of the condition (constant or non-constant pain, disturbed sleep, limitation and restriction level according to the ODI results), the most limited plane of motion (as the prescribed exercises were primarily carried out in the planes of motion that showed limited mobility or aberrant movements) and the quality of exercise execution. The physiotherapist adjusted the difficulty level of the exercises in order to prompt maximal effort without jeopardizing the quality of the movements. Exercises were paused or stopped when fatigue led to the deterioration of the quality of movements. See the summary table of principles on exercise selection and progression in the Additional file 1. Individual interventions such as manual therapy and other physiotherapy treatment modalities were not performed during the course of the study.

### Data analysis

Based on their favorable (50% improvement in the ODI score) or unfavorable outcome following participation in the program, subjects were classified into two subgroups. A first screening of the potential prognostic factors of outcome was done using univariate analyses by looking at statistical differences between groups on characteristics, questionnaires and physical examination data at baseline (Tables 1, 2 and 3). Independent t-tests (continuous variables) and chi-square tests (categorical variables) were used. A potential prognostic factor was retained when the *p*-value was lower or equal to 0.1.

**Table 1:** Baseline participants’ characteristics

Variables	All Subjects (*N* = 85)	Favorable outcome (*N* = 40)	Unfavorable outcome (*N* = 45)
Age (years)	37.22 ± 9.35	36.51 ± 10.40	37.83 ± 8.38
Gender (% men)	88.2	85.0	91.1
Weight (kg)	86.27 ± 18.57	83.82 ± 16.78	88.46 ± 19.96
Height (m)	1.74 ± 0.08	1.74 ± 0.08	1.74 ± 0.07
Body mass index (kg/m^2^)	27.93 ± 5.93	27.51 ± 4.65	28.29 ± 6.90
Number of months in the army	158.30 ± 111.58	147.25 ± 112.34	168.34 ± 111.21
History of LBP (%)	74.1	70.0	77.8
Number of months since last onset of LBP	16.28 ± 32.19	12.12 ± 20.34	19.69 ± 39.38
Number of treatments received before first evaluation*	4.94 ± 5.81	3.14 ± 3.81	6.85 ± 6.92
Referred pain in lower limb (%)	31.8	22.5	40.0
Work restriction (%)*	51.8	40.0	62.2
Work restriction of 6 months or more (%)*	30.6	15.0	44.4
Numbness and tingling sensation (%)	23.5	22.2	26.7
Prone instability test (%)	66.2	71.4	61.1
Use of antidepressants (%)*	15.3	5.0	24.4
Use of nonsteroidal anti-inflammatories (%)	29.4	32.5	26.7
Pain in sitting position (VAS 0–100)	77.6	77.5	77.8
Pain in lying position* (VAS 0–100)	37.5	25.0	48.9
Pain in standing position (VAS 0–100)	84.7	75.0	93.3
Pain during walking (VAS 0–100)	40.0	32.5	46.7
Pain when coughing or sneezing (VAS 0–100)	28.2	25.0	31.1
Tingling or numbness in the lower limbs (VAS 0–100)	24.7	20.0	26.7

*Significant difference between the favorable outcome group and unfavorable outcome group (*p* < 0.10; univariate analyses). Continuous variables are shown as Mean ± 1 Standard Deviation, while categorical variables are shown as %

**Table 2:** Baseline participants’ questionnaire scores

Questionnaires	All Subjects (N = 85)	Favorable outcome (N = 40)	Unfavorable outcome (N = 45)
FABQ Work subscale (0–42)*	21.04 ± 10.75	16.63 ± 9.43	25.05 ± 10.38
FABQ Physical Activity subscale (0–24)	13.23 ± 5.03	12.57 ± 5.08	13.8 ± 5.0
Worst LBP perceived in the last 48* hours (1 to 10 scale)	4.65 ± 1.94	4.28 ± 1.94	4.98 ± 1.90
Mean LBP perceived in the last 48 h (1 to 10 scale)	2.96 ± 1.30	2.73 ± 1.26	3.18 ± 1.31
Modified Oswestry Disability Index (0–100)	32.1 ± 12.1	32.1 ± 12.7	32.2 ± 11.8

*Significant difference between the favorable outcome group and unfavorable outcome group (*p* < 0.10; univariate analyses). Continuous variables are shown as Mean ± 1 Standard Deviation, while categorical variables are shown as %

**Table 3:** Performance in the clinical tests at baseline

Variables	All Subjects (N = 85)	Favorable outcome (N = 40)	Unfavorable outcome (N = 45)
Mean distance fingertip to ground in trunk flexion (cm)	10.67 ± 10.87	10.96 ± 10.51	10.41 ± 11.29
Mean distance fingertip to ground in trunk lateral flexion (cm)	48.18 ± 4.52	48.28 ± 4.22	48.08 ± 4.81
Trunk lateral flexion discrepancy (R-L, cm)	2.37 ± 2.27	2.21 ± 1.87	2.51 ± 2.59
Worst trunk lateral flexion (cm)	46.99 ± 4.60	47.17 ± 4.38	46.83 ± 4.84
Mean trunk extension (deg)	24.23 ± 9.91	24.82 ± 9.79	23.71 ± 10.10
Mean hip internal rotation (deg)	30.84 ± 9.54	5.97 ± 7.25	5.97 ± 4.68
Hip internal rotation discrepancy (R-L, deg)	7.54 ± 6.33	28.32 ± 9.23	27.42 ± 10.17
Worst hip internal rotation (deg)	27.84 ± 9.69	31.31 ± 9.02	30.41 ± 10.06
Mean hip external rotation (deg)	45.12 ± 10.87	45.76 ± 11.26	44.55 ± 10.59
Hip external rotation discrepancy (R-L, deg)	5.97 ± 5.99	7.22 ± 6.65	7.82 ± 6.09
Worst hip external rotation (deg)	41.35 ± 11.81	42.15 ± 12.53	40.64 ± 11.23
Mean hip flexion (deg)	111.08 ± 8.87	111.33 ± 10.38	110.84 ± 7.64
Hip flexion discrepancy (R-L, deg)	4.36 ± 3.24	4.55 ± 3.13	4.20 ± 3.36
Worst hip flexion (deg)	106.45 ± 11.04	108.47 ± 11.32	104.66 ± 10.58
Mean hip extension (deg)	11.15 ± 11.05	11.07 ± 11.95	11.22 ± 10.31
Hip extension discrepancy (R-L, deg)	3.08 ± 2.76	2.90 ± 2.53	3.24 ± 2.97
Worst hip extension (deg)	9.61 ± 10.99	9.62 ± 12.04	9.60 ± 10.10
Mean lateral plank (sec)	65.72 ± 35.46	66.79 ± 30.58	64.76 ± 39.61
Lateral plank discrepancy (R-L, sec)	14.00 ± 12.74	14.62 ± 14.07	13.45 ± 11.56
Worst lateral plank (sec)	58.57 ± 33.75	57.86 ± 26.26	59.14 ± 39.11
Mean score on Biering-Sorensen test (sec)	84.19 ± 44.12	95.10 ± 47.09	74.48 ± 39.33
Mean score on McGill abdominal test (sec)	101.96 ± 56.29	112.07 ± 58.65	92.97 ± 53.15
Mean Straight Leg Raise (deg)	72.85 ± 10.95	72.55 ± 10.59	73.12 ± 11.36
Straight Leg Raise discrepancy (R-L, deg)	4.76 ± 4.78	4.40 ± 4.04	5.08 ± 5.38
Worst Straight Leg Raise (deg)	70.47 ± 11.46	70.35 ± 10.77	70.57 ± 12.16

sec: second, deg.: degree, cm: centimeter. All measurements are shown as Mean ± 1 Standard Deviation. None of the clinical variables showed a significant difference (univariate analyses)

Before entering the potential prognostic factors into a multiple logistic regression, the Kaiser-Meyer-Olkin (KMO) index was calculated on the set of variables that included all the potential prognostic factors and the group variable. The KMO index and measures of sampling adequacy (MSA) were used to determine collinearity. When the KMO index is greater or equal to 0.6, the logistic regression may be performed with all the potential prognostic factors. When the MSA are below 0.6, the associated potential prognostic factor must be removed.

The first multiple logistic regression included all valid prognostic factors. Considering that the useful information brought by one prognostic factor may be entirely offered by another, prognostic factors with very high *p*-values (*p* > .50) were removed from the model even though they had significant *p*-values with the univariate tests. The aim was to obtain the most efficient prognostic factors. A second multiple logistic regression was then calculated on a model that included only prognostic factors that had *p*-values below 0.50. From a clinical perspective, continuous factors are often impractical. Discrete criteria are better suited to clinical use. For this reason, the continuous variables retained by the second model were dichotomized based on thresholds determined by recursive partitioning analysis (SPSS, proc. TREE). Then, a third and final multiple logistic regression was calculated with the dichotomized version of the set of prognostic factors from the second model. The anti-image and inverse of the correlation matrix, drawn from factor analyses with and without the dependent variable, were used to test for collinearity between variables included in the model. Finally, the sensitivity, specificity and the positive and negative likelihood ratios (LR+ and LR-, respectively) were determined for each variable and for the overall regression model. All analyses were conducted with SPSS software (Version 23; IBM SPSS Statistics for Mac. Armonk, NY: IBM Corp) except for the sensitivity, specificity and likelihood ratios (with 95% confidence intervals) which were estimated with the package epiR (Tools for the Analysis of Epidemiological Data, a package available for the 3.3.1 version of the R statistical software).

## Results

Of the 104 individuals that were recruited, nineteen (18.3%) did not complete the 6-week program or did not take part in the final evaluation. Therefore, a constant sample of 85 participants (81.7%) was included in all analyses. The reasons given by the individuals for dropping out of the study were: difficulties in coming to the Valcartier Health Centre due to military duties (*n* = 2), medical reasons (*n* = 1), new employment outside of the military (n = 2), prolonged absence for a military exercise or mission (*n* = 3) and personal or unknown reasons (*n* = 11). The individuals who dropped out had higher scores on the numerical pain rating scale evaluating worst and mean LBP perceived in the last 48 h when compared to the participants included in the final analyses (*p* < 0.05). No other differences were found. Subjects retained for analyses participated in 14.4 ± 2.6 sessions on average during the 6-week program (a mean of 2.34 sessions/week).

The mean baseline ODI score for the whole sample was 32.1 ± 12.1, considered as a moderate disability level [26]. Forty participants (47%) were categorized as having favorable outcome based on the ODI criterion, and 45 participants (53%) as unfavorable. The mean ODI change was 23.9 ± 11.0 for the group with favorable outcome and 3.4 ± 10.3 for the group with unfavorable outcome.

Tables 1, 2 and 3 present the clinical variables at baseline for the whole sample, as well as for both groups with favorable/unfavorable outcome. Notably, 74 % of the participants had previous episodes of LBP, and the time elapsed since the last episode varied from 1 to 200 months (mean 16.3 ± 32.2 months). Thirty-two percent (*n* = 27) had referred pain to the lower limbs. Finally, 51.8% of the participants (*n* = 44) had work restrictions.

The univariate tests identified seven potential prognostic factors with a KMO index of .692. As all MSA were above .600, no potential prognostic factors needed to be removed. The first multiple logistic regression indicated that two prognostic factors (work restrictions because of LBP and worst LBP perceived in the last 48 h) brought no unique information, with *p*-values respectively of .843 and .813, and were thus removed from the model. The second set of five factors with the group variable had a KMO index of .635. Although two prognostic factors had a MSA value very slightly below .6, all were kept in the model as these MSA were above .6 when the seven prognostic factors were used. [40] Furthermore, when the prognostic factors were dichotomized (see below), these MSA rose respectively to .60 and .62, and all five MSA were above or equal to .6. The model of the second multiple logistic regression had a highly significant (*p* = .00011) moderate capacity (Nagelkerke R^2^ = .412) to predict the program outcome (favorable outcome: 86% correct; unfavorable outcome: 70% correct; global: 78% correct). This set of five variables included: (1) “no pain in lying down” [dichotomous], (2) “no use of antidepressants” [dichotomous], (3) “FABQ Work subscale” [continuous], (4) “number of treatments received before the first evaluation” [continuous], and (5) “no work restriction of 6 months or more” [dichotomous]. Individually, only variables 1 and 4 had *p*-values below .05 (respectively of .017 and .030). Since we were at the hypothesis-setting step of CPR development and since it is possible that beyond our sample these variables contain unique information that can predict the favorable or unfavorable outcomes, we decided to continue with the full set of five variables.

The FABQ Work subscale was dichotomized with the help of a recursive partitioning technique that indicated an optimal cutoff point of 22.5. Then, the potential dichotomized prognostic factor of a favorable outcome was set at a score below 22.5 on the FABQ Work subscale. The recursive partitioning technique did not successfully dichotomize the number of previous treatments. Therefore, this variable was dichotomized based on the threshold that led to the model yielding the best accuracy in predicting outcome. A criterion of 4 previous treatments or fewer was set as a potential dichotomized prognostic factor of favorable outcome.

The results of the third and final multiple logistic regression are shown in Tables 4 and 5. The model has a significant (*p* = .00004) moderate capacity (Nagelkerke R^2^ = .370) to predict the program outcome (favorable outcome: 78% correct; unfavorable outcome: 80% correct; global: 79% correct). Furthermore, as the five variables had an odds ratio above 2.0 in this final regression, we decided to retain all variables in the final model. Even though the Nagelkerke R^2^ may be lower, the outcome classification appears almost identical if not very slightly better. There was no sign of multicollinearity between variables included in the model. As seen in Table 4, only the first variable has a *p*-value below .05 (*p* = .017; Odds ratio = 3.7 [1.3–10.6]). It is also noteworthy that two variables have *p*-values of .061: (a) “no use of anti-depressants” (Odds ratio: 5.2 [0.9–29.4]), and (b) “FABQ Work below 22.5” (Odds ratio: 2.9 [0.9–8.6]).

**Table 4:** Predictive capacity of individual variables obtained in the multiple logistic regression

Variables	Sig.	Sensitivity	Specificity	LR+	LR-	Odd Ratio
No pain in lying down	0.017	0.75 [0.58–0.87]	0.49 [0.34–0.64]	1.47 [1.05–2.06]	0.51 [0.29–0.92]	3.65 [1.3–10.6]
No use of antidepressants	0.061	0.95 [0.82–0.99]	0.24 [0.13–0.40]	1.26 [1.05–1.51]	0.20 [0.05–0.90]	5.2 [0.9–29.4]
FABQ Work < 22.5	0.061	0.73 [0.56–0.85]	0.67 [0.51–0.80]	2.18 [1.38–3.43]	0.41 [0.24–0.70]	2.9 [0.9–8.6]
Number of previous treatments < 5	0.144	0.68 [0.51–0.81]	0.58 [0.42–0.72]	1.60 [1.07–2.39]	0.56 [0.35–0.91]	2.2 [0.8–6.3]
Work restriction < 6 months	0.161	0.85 [0.69–0.94]	0.44 [0.30–0.60]	1.53 [1.14–2.05]	0.34 [0.15–0.75]	2.48 [0.7–8.8]

Numbers in square brackets represent the 95% confidence interval

**Table 5:** Predictive capacity according to the number of criteria present

Number of Prognostic factors	Sensitivity	Specificity	LR+	LR-
One or more	1.00 [0.89–1.00]	0.02 [0.001–0.13]	1.02 [0.98–1.07]	N/A
Two or more	0.95 [0.82–0.99]	0.18 [0.09–0.33]	1.16 [0.99–1.35]	0.28 [0.06–1.33]
Three or more	0.90 [0.75–0.96]	0.49 [0.34–0.64]	1.76 [1.30–2.39]	0.20 [0.07–0.54]
Four or more	0.78 [0.61–0.89]	0.80 [0.65–0.90]	3.88 [2.11–7.12]	0.28 [0.16–0.50]
Five	0.33 [0.19–0.49]	0.93 [0.81–0.98]	4.88 [1.50–15.88]	0.72 [0.58–0.90]

Numbers in square brackets represent the 95% confidence interval

In sum, for a patient or client presenting with all five criteria, the prognostic factors have low sensitivity and high specificity. The sensitivity improves to 0.78 for individuals presenting at least four of the five criteria. However, this increase in sensitivity comes at the expense of a decrease in specificity. Table 6 reports the prediction rate of a favorable outcome according to the number of criteria fulfilled. The satisfaction of four out of five criteria appears to be the best compromise between sensitivity and specificity.

**Table 6:** Prediction rate of a favorable outcome according to the number of criteria fulfilled

Number of variables	0	1	2	3	4	5	0 to 3	4 or 5
Favorable outcome	0.0%	22.2%	12.5%	26.3%	75.0%	81.3%	20.0%	77.5%
Unfavorable outcome	100%	77.8%	87.5%	73.7%	25.0%	18.7%	80.0%	22.5%

## Discussion

This study identified five variables of a favorable outcome in patients with LBP who participated in a multi-station full-body supervised exercise program. Clinicians may anticipate a favorable outcome when their initial assessment identifies 4 or 5 variables included in our model. On the other hand, a less favorable outcome is expected if 3 or fewer variables are present, since the expected failure rate would be 80%. It is to be noted that these findings were established for a 50% improvement on the ODI and different variables would likely have been obtained had a different ODI threshold or other outcomes been used. Furthermore, the LR+ (3.9) and LR- (0.28) were relatively low [35], suggesting that the predictive capability of the model is limited. In comparison, Hicks et al. [15] found similar LR+ (LR+ 4.0), but higher LR- (LR- 0.18), with their preliminary CPR. Rabin et al. could not, however, validate this preliminary CPR in a subsequent study [13]. In contrast, Stolze et al. [23] reported a LR+ of 10.6 (95% confidence interval [CI]: 3.52, 32.14) in a CPR deviation study created to generate potential treatment effect modifiers of pilates exercises.

Three of the five variables included in our model had already been identified as prognostic factors of favorable outcomes for individuals with LBP in previous studies. We found that a score below 22.5 on the FABQ work subscale predicted a favorable outcome, which is in agreement with previous studies in which fears and beliefs about work were associated with poor recovery in patients with work-related LBP [41]. The FABQ work score was not identified as a predictor in CPRs developed by Hicks et al. [15] and Stolze et al.[23]. However, a score of less than 19 on the FABQ work subscale was considered a treatment effect modifier in the CPR developed by Flynn et al. [22], later validated by Childs et al., [42] whose aim was to identify patients likely to benefit from lumbar manipulation. As in the current study, participants in Flynn and Child’s studies were recruited from care facilities within the military, suggesting that fear-avoidance beliefs about military tasks may be of particular concern in soldiers with LBP. We also found that patients who do not use antidepressant medication were more likely to have a better outcome. This finding suggests that patients requiring antidepressants had less favorable outcomes, an observation which concords with the literature showing that patients with LBP in a depressive state are predisposed to longer recovery time and to the development of chronicity [43, 44]. A third prognostic factor of outcome in our model was “no work restriction of six months or more”. In the military context, a work restriction of fewer than six months should be interpreted as a temporary modification of regular duties for a non-chronic health condition, in contrast to a six-month medical category which is attributed to people for whom slow recovery or permanent health problems are anticipated. This finding is in agreement with previous studies that identified prolonged absence from work and the number of days of reduced activity as predictors of slower recovery in patients with LBP [41, 43]. “No pain when lying down” and “having had fewer than five physiotherapy treatments before the baseline evaluation”, that were both identified as prognostic factors in our model, have, to our knowledge, never been associated with the outcome of LBP. It is to be noted that the cutoff criteria for two of the prognostic factors (FABQ Work subscale and number of previous treatments) are actually tied to our sample and must be confirmed by future studies.

This single-arm study was the preliminary step of a process aiming to develop a CPR that can be used to identify patients most likely to benefit from the proposed exercise program. Three of the five variables identified in our final model are generally recognized as prognostic factors in people with LBP and only one of these has been validated as a treatment effect modifier within a CPR aiming to predict patients likely to benefit from lumbar manipulation. The methodological approach used in our study was deliberately liberal. To minimize the possibility of missing potential prognostic factors, we set the *p* value for retaining variables at 0.1 and, as suggested in hypothesis-setting studies, [20] we did not perform corrections for multiple comparisons. Investigating a high number of variables (more than 50) in a relatively small sample, as we did in the present study, increases the likelihood of finding significant associations by chance (type 1 error), which can be considered a limitation. However, only one prognostic factor was below the generally accepted level of significance of 0.05 and our final model has only a limited predictive capability. Thus, before continuing to the validation step, our results need to be confirmed in a hypothesis-testing study in which a limited number of a priori hypotheses will be tested and appropriate adjustments for multiple comparisons will be made. Any subsequent validation study should also be conducted with non-military groups (broad validation), as the present results may not be generalizable to the greater population. On the other hand, the well-standardized multivariate protocol and the acceptable dropout rate represent strengths of this study. Finally, the targeted sample size of 90 participants to be included in the statistical analyses was not met, as only 85 of the 104 participants took part in both evaluation sessions.

## Conclusion

The present study established five variables to identify patients most likely to have a favorable outcome, regardless of their participation in the exercise program. Careful use of these variables is mandatory for clinical purposes as this study is at the early stage of CPR development. Future validation studies should be carried out with other populations to confirm this CPR and subsequently, to verify whether some of these factors may be considered treatment effect modifiers.

## Additional file

Additional file 1: Illustrations of the exercises included in the multi-station program. (DOCX 923 kb)

## Abbreviations

CI: Confidence interval
CPR: Clinical prediction rule
FABQ: Fear-avoidance beliefs questionnaire
ICC: Intraclass correlation coefficient
KMO: Kaiser-Meyer-Olkin
LBP: Low back pain
LR: Likelihood ratio
MSA: Measure of sampling adequacy
NPRS: Numeric pain rating scale
ODI: Modified Oswestry disability index
ROM: Lumbar range of motion
TBC: Treatment-based classification
