# Supplementary material for: Prognostic factors of a favorable outcome following a supervised exercise program for soldiers with sub-acute and chronic low back pain
Source: BMC Musculoskelet Disord. 2018 Apr 2;19:95. doi: 10.1186/s12891-018-2022-x (PMC5879551; doi:10.1186/s12891-018-2022-x)
Supplement: Supplementary file 1 — Illustrations of the exercises included in the multi-station program. (DOCX 923 kb) [file 12891_2018_2022_MOESM1_ESM.docx]

**Illustrations of the exercises included in the multi-station program**

**STATION 1: HIP STRENGTHENING AND CONTROL**

**1.1 Cat/Camel Back (1 X 8-10 repetitions)** **1.2 Hip Flexion (2 X 5 repetitions)**


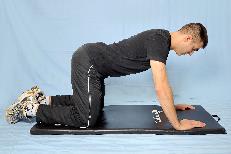
**⇒**
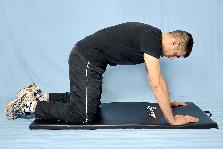

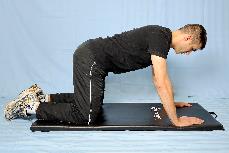
**⇒**
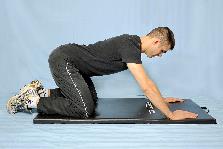


**1.1 (A)**  **1.1 (B)**  **1.2 (A)** **1.2 (B)**

**1.3 Pivot (2 X 5 repetitions)**


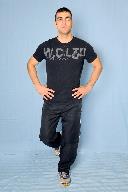
 **⇒**
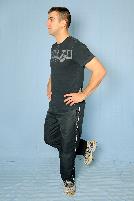

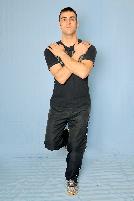

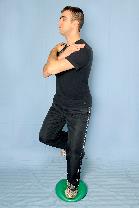

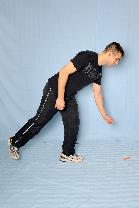
 **⇒**
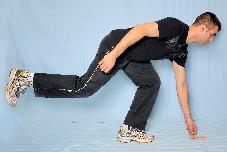


**1.3.1 (A)** **1.3.1 (B)** **1.3.2** **1.3.3** **1.3.4 (A)** **1.3.4 (B)**

**1.4 Sit to Stand Transfer (2 X 5 repetitions)**


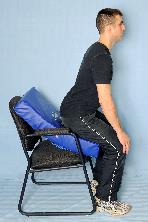

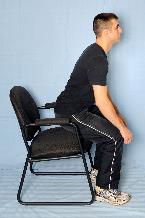

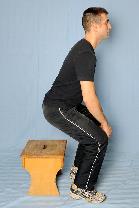


**1.4.1** **1.4.2**  **1.4.3**

**1.5 ″ Japanese Stick ″ (2 X 30 seconds)**


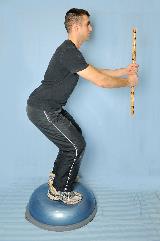

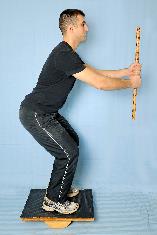

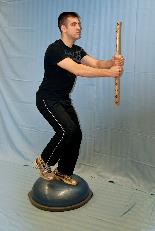

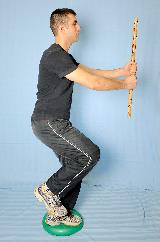

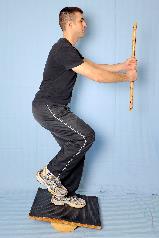


**1.5.1** **1.5.2** **1.5.3** **1.5.4**  **1.5.5**

**1.6 Balance on a stool (2 X 30 sec)** **1.7 Targets on the wall (2X 30 sec)**


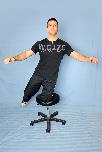

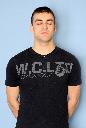

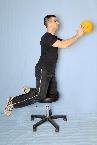

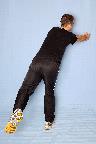

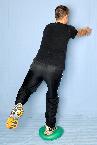

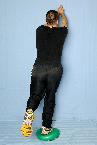


**1.6.1** **1.6.2** **1.6.3** **1.7.1** **1.7.2** **1.7.3**

**1.8 Hip Strengthening (hold 10 seconds, 2 X 10 repetitions)**


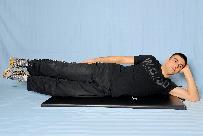

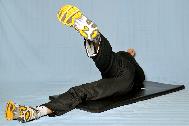

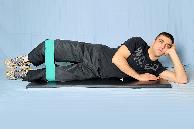

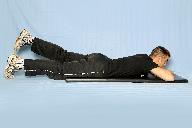


**1.8.1** **1.8.2** **1.8.3**  **1.8.4**

**Station 2: THE SQUAT AND ITS VARIANTS**

**2.1 Half-squat (hold 20-30 seconds, 3 sets)**


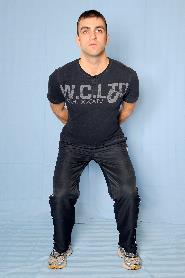

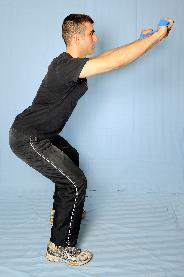

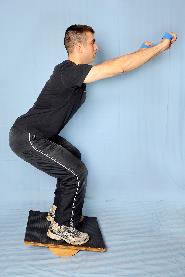

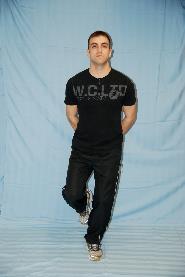


**2.1.1**  **2.1.2**  **2.1.3** **2.1.4**


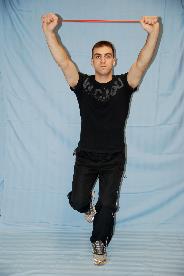

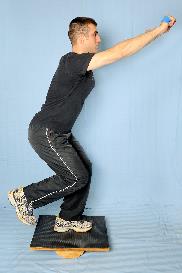

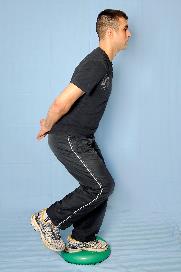


**2.1.5** **2.1.6** **2.1.7**

**2.2 The Star (2 X 30 seconds)**


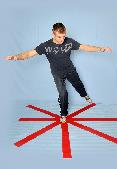

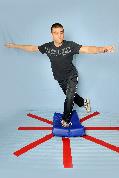

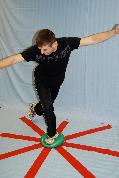


**2.2.1** **2.2.2** **2.2.3**

**2.3 Lunge (2 X 10 repetitions)**


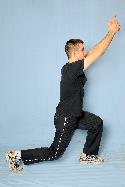

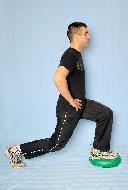

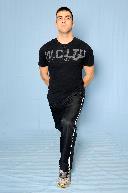

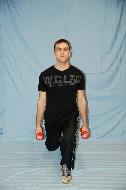

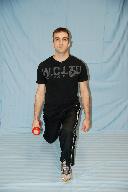

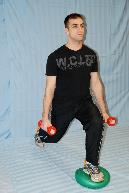

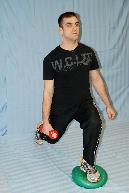


**2.3.1** **2.3.2** **2.3.3** **2.3**.4 **2.3.5** **2.3.6** **2.3.7**

**2.4 Shuttle   (2 X 8-12 repetitions)**


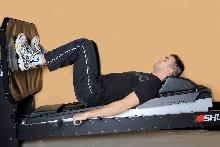
 **⇒**
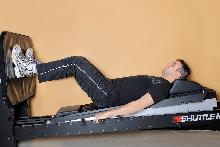


**2.4.1 (A)** **2.4.1 (B)**


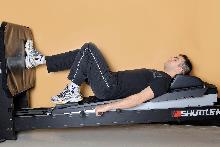

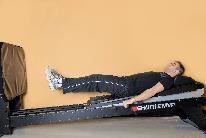

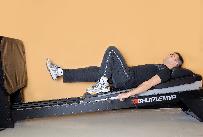


**2.4.2 2.4.3** **2.4.4**

**2.5 Jumps (2 X 10 repetitions)**

**
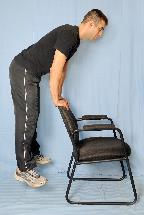

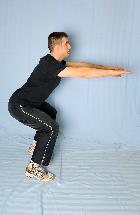

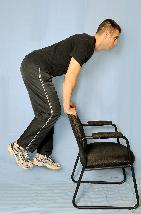

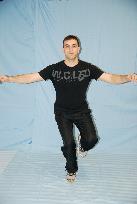

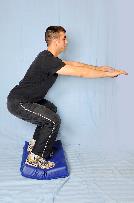

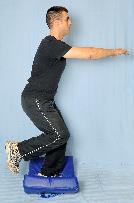
**

**2.5.1 2.5.2 2.5.3 2.5.4 2.5.5 2.5.6**

**Station 3: ELASTIC BANDS AND THE BODYBLADE**

**3.1 Static position (hold 30 seconds, 2 sets on each side)**

**
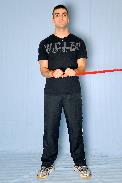

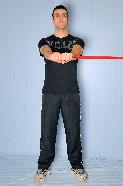

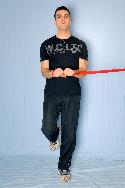

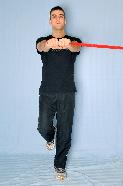

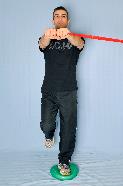

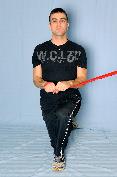

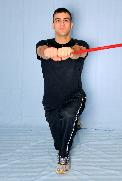
**

**3.1.1 3.1.2 3.1.3 3.1.4 3.1.5 3.1.6 3.1.7**

**3.2 Dynamic movement of the upper limbs (2 X 10 repetitions on each side)**

**
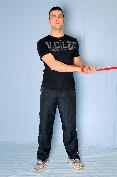
 ⇒
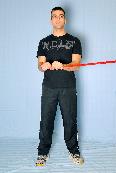
 ⇒
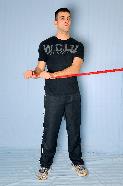

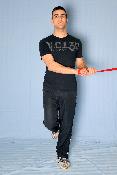
 ⇒
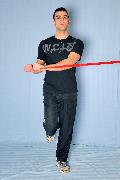
**

**3.2.1 (A) 3.2.1 (B) 3.2.1 (C) 3.2.2 (A) 3.2.2 (B)**

**3.3 Dynamic rotation of the trunk (2 X 10 repetitions on each side)**

**
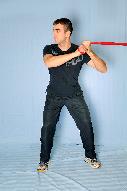
 ⇒
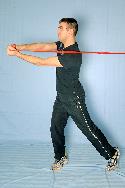

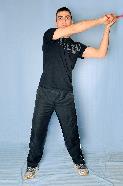
 ⇒
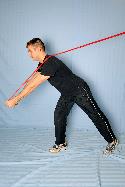

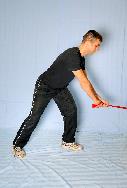
 ⇒
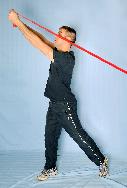
**

**3.3.1 (A) 3.3.1 (B) 3.3.2 (A) 3.3.2 (B) 3.3.3 (A) 3.3.3 (B)**

**3.4 Bodyblade (3 X 20 seconds)**

**
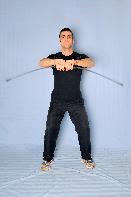

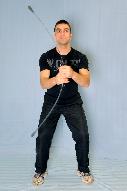

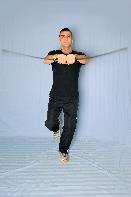

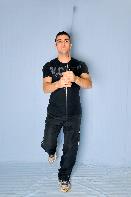
**

**3.4.1 3.4.2 3.4.3 3.4.4**

**STATION 4: ABDOMINAL PLANKS AND THEIR VARIANTS**

**4.1 Push up position (3 X 20-30 seconds)**


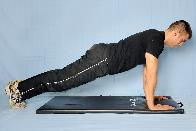

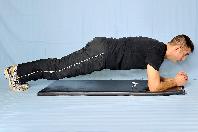

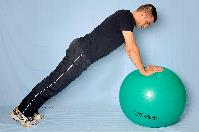

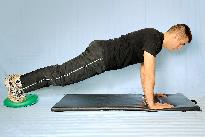


**4.1.1** **4.1.2** **4.1.3** **4.1.4**


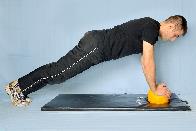

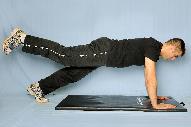

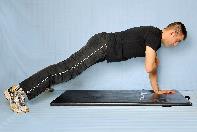

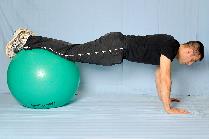


**4.1.5**  **4.1.6**  **4.1.7** **4.1.8**


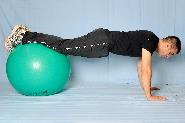
 **⇒**
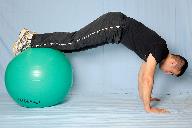

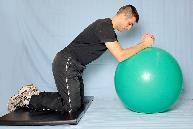
 **⇒**
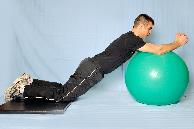


**4.1.9 (A)** **4.1.9 (B)** **4.1.10 (A)** **4.1.10 (B)**


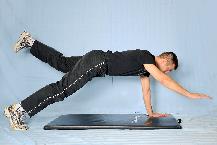


**4.1.11**

**4.2 Pivot in the Push up position (2 X 20-30 seconds, alternate sides)**


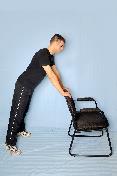
 **⇒**
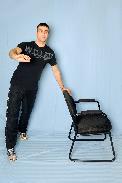

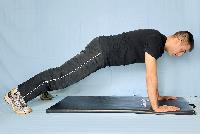
 **⇒**
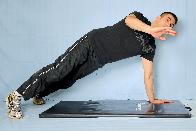
 **
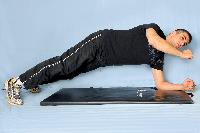
**

**4.2.1 (A) 4.2.1 (B) 4.2.2 (A) 4.2.2 (B) 4.2.3**

**4.3 Sprint start from prone position (2 X 8-10 repetitions)**

**
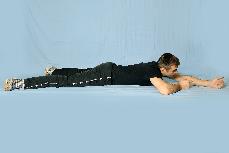

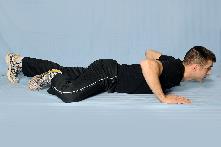

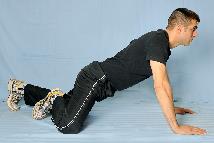

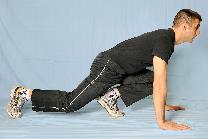
**

**4.3 (A) 4.3 (B) 4.3 (C) 4.3 (D)**

**STATION 5: ABDOMINAL STRENGTHENING**

**5.1 « Curl up » (2 X 10 repetitions, hold position for 8 seconds)**

**5.1.1 5.1.2 5.1.3**

**5.2 Lateral plank (2 X 10 repetitions, hold position for 8 seconds)**

**5.2.1 5.2.2 5.2.3 5.2.4**

**5.3 « Curl up » on a Swiss Ball (3 X 20-30 seconds)**

**5.3.1 5.3.2 5.3.3**

**5.4 Catching a medicine ball  (3 X 20 seconds)**

**5.4.1 5.4.2 5.4.3**

**STATION 6: BACK EXTENSOR STRENGTHENING**

**6.1 « Bird Dog » (2 X 10 repetitions, hold position for 8 seconds)**

**6.1.1 6.1.2 6.1.3**

**6.2 Strengthening on a Swiss Ball (2 X10 repetitions, hold position for 8 seconds)**

**6.2.1 6.2.2 6.2.3**

**6.2.4 6.2.5**

**6.3 Lower limb extension (2 X 10 repetitions, hold position for 8 seconds)**

**6.3.1 6.3.2 6.3.3**

**6.3.4 6.3.5**

**6.4 Bridge (2 X 10 repetitions, hold position for 8 seconds)**

**6.4.1 6.4.2 6.4.3 6.4.4**

**6.5 Roman Chair (2-3 X hold position for one minute)**

**6.5.1 6.5.2 6.5.3 6.5.4**

**STATION 7: LIFTING TECHNIQUES**

**7.1 Deadlift (2-3 X 8-10 repetitions)**

**⇒ ⇒ ⇒ ⇒**

**7.1.1 (A) 7.1.1 (B) 7.1.1 (C) 7.1.2 (A) 7.1.2 (B) 7.1.2 (C)**

**7.2 Weight transfers and walking with a load (2 X 5 repetitions)**

**⇒ ⇒ ⇒ ⇒**

**7.2.1 (A) 7.2.1 (B) 7.2.1 (C) 7.2.2 (A) 7.2.2 (B) 7.2.2 (C)**

**7.3 Unilateral lift (2 X 10 repetitions)**

**⇒ ⇒**

**7.3.1 (A) 7.3.1 (B) 7.3.1 (C) 7.3.2**

**⇒ ⇒ ⇒ ⇒**

**7.3.2 (A) 7.3.2 (B) 7.3.2 (C) 7.3.3 (A) 7.3.3 (B) 7.3.3 (C)**

**7.4 Lifting a Rucksack (1 X 5 repetitions)**

**⇒ ⇒**

**7.4.1 (A) 7.4.1 (B) 7.4.1 (C)**

**7.5 Casualty drag (2 X 5 repetitions)**

**⇒**

**7.5.1 (A) 7.5 1 (B)**

| **Exercises parameters and selection** |
| --- |
| Basic principles applied for all exercises   - A natural lumbar lordosis should me maintained in all times, regardless of the weight or external load imposed on the body - A large variety of exercises should be completed - Focus should be kept on the quality rather than the quantity of movements   The selection of exercises and initial level of difficulty is determined according to 3 criteria:   1. The severity of the condition (pain at rest, disturbed sleep, level of limitation and restriction) 2. The most limited plane of motion: the prescribed exercises should be primarily carried out in the planes of motion that showed limited mobility or aberrant movements 3. The quality of exercises execution: maximal effort during exercises should not jeopardizing the quality of the movements. |
